# David Hörburger: EKG-Training mit QR^+^ – 70 wichtige EKGs mit klinischem Hintergrund

**DOI:** 10.3205/zma001866

**Published:** 2026-09-15

**Authors:** Sigrid Harendza

**Affiliations:** 1 Universitätsklinikum Hamburg-Eppendorf, III. Medizinische Klinik, Hamburg, Germany

## Bibliographical details

David Hörburger

**EKG-Training mit QR** **^+^** **– 70 wichtige EKGs mit klinischem Hintergrund**

Publisher: Hogrefe-Verlag

Year of publication: 2026, 160 pages, price: € 40,00

ISBN: 978-3-456-86303-0

## Review

This book is fun right from the moment you unpack it. The A4 landscape format somehow immediately gives you the feeling that you are holding real ECGs in your hands. So, I dive right in and open the book at a page somewhere in the middle. With my heart pounding a little, I look at the ECG on the left-hand page. Hm, it looks like S1/Q3 and right bundle branch block, hence, a suspected pulmonary embolism. Then I read the corresponding case on the right-hand page and look at the listed vital signs. They fit well with my suspected diagnosis. Now I’m curious, so instead of writing down my first impression, my interpretation, and my uncertainties in the margin on the right, I immediately scan the QR code next to it. Above the QR code the word “solution” is written, and this lets me try out the part of the book hidden in the QR^+^ on the title of the book. A sense of relief washes over me: my ECG interpretation and preliminary diagnosis were correct. But oh, there is so much to discover here: a complete ECG interpretation, a detailed case commentary, and a video stream with additional clinical information on diagnosis and treatment. Now the time has probably come to explore the educational concept behind this book, which is intended to serve as an atlas to help learners apply the theoretical principles of ECG interpretation.

From the preface, I gather that the author developed the atlas as a project of his part-time Master of Medical Education program at the University of Bern. This suggests a truly praxis-oriented product, and that is indeed the case. The fist spread concisely explains how to use the atlas. Each ECG is presented within the context of a clinical case. The ECG should be interpreted in the context of the clinical symptoms described. The workflow consists of five steps:

1. Reading the case vignette,
2. examining the ECG and making a note of one’s first impression,
3. interpreting the ECG (exact instructions what to do, e.g., determining the cardiac axis, measuring the PQ interval, etc., are provided),
4. documenting uncertainties, and
5. reading the correct interpretation, diagnosis, and case commentary in the electronic supplementary material.

To access this material, a code is provided on the instruction page when the atlas is purchased. Then comes the real highlight. On the download page, that is, the QR^+^, one finds a video with detailed case interpretations, additional tips, information about potential pitfalls, and treatment recommendations. Finally, there are references to further topics one can explore in greater depth using ECG teaching material of one’s choice. So now I could start once again, working through the atlas systematically. To my delight, as I turn the page, I notice that it does not start with the first case right away, but that the actual exercises are preceded by a concise theoretical section less than ten pages long, covering everything from ECG physiology to ECG taking and the normal values for the individual ECG segments. This section can be quickly skimmed through as a brief refresher and, according to the note in the preface, is not intended to replace learning the theoretical fundamentals of the ECG. 

Instead of working systematically with the atlas at this point, I am taking another, somewhat closer look at the QR^+^ part. It starts in a very welcoming way with a note inviting users to share feedback or suggestions, and an email link is provided for this purpose. This is followed by an overview of the 70 cases, each of which can be clicked on individually to access the descriptions and interpretations. For the video streaming, the start time for each ECG is listed, but you have to manually adjust the video to that point – at least with my setup. It would be the icing on the cake if one could simply click to jump directly to the right spot in the video. From an educational point, it is very well done that the ECGs in the video are organized according to a list of categories that is also displayed (normal ECG, arrhythmias, extracardiac causes, etc.), so it is also possible to work through the ECGs in this order if one wants to learn according to this classification. The explanations, presented in the form of a narrated video, are very informative and focused; the speaking pace might be a bit fast for beginners, but one can always halve the speed if one wants to, or pause the video along the way. This makes working with this atlas in general and with the QR^+^ section in particular very easy to adapt to one’s own learning preferences, needs, and pace. Thanks to these flexible ways of working with the atlas and its quiz-based or systematic approach, the learning resource seems suitable for use both in medical school and in postgraduate medical training.

## Author’s ORCID

Sigrid Harendza: [0000-0002-7920-8431]

## Competing interests

The author declares that she has no competing interests.

